# Proximal versus distal adductor canal blocks for total knee arthroplasty: A protocol for randomized controlled trial

**DOI:** 10.1097/MD.0000000000019995

**Published:** 2020-05-29

**Authors:** Quan Wang, Yijun Zhang, Jingyu Du, Xiangjin Lin

**Affiliations:** Department of Orthopedics, The First Affiliated Hospital of Zhejiang University, Hangzhou City, China.

**Keywords:** distal adductor canal block, pain control, proximal adductor canal block, randomized controlled trial, study protocol, total knee arthroplasty

## Abstract

**Background:**

Currently, there remains a paucity of literature about the efficiency of proximal adductor canal block (PACB) versus distal adductor canal block (DACB) for pain management after total knee arthroplasty (TKA). The purpose of this study is to perform a randomized controlled trial to compare the efficiency of PACB versus DACB for early postoperative pain treatment after TKA.

**Methods:**

This study is a 2-arm, parallel-group, randomized controlled trial that is conducted at a single university hospital in China. Subjects presenting for unilateral TKA are randomized in a 1:1 ratio to either a PACB or DACB group. The primary outcome of this noninferiority study is opioid consumption within the first 24 hours following surgery. Secondary outcomes include quadriceps strength, pain scores, distance ambulated, and patient satisfaction. Continuous variables are compared using Student *t* test.

**Results:**

This clinical trial is expected to provide evidence of whether the PACB and DACB provide similar analgesia after TKA.

**Trial registration:**

This study protocol was registered in Research Registry (researchregistry5440).

## 1 Introduction

Total knee arthroplasty (TKA) is very commonly performed in western countries. Major goals following TKA include providing sufficient postoperative pain treatment to assist early physical therapy and allowing patients to return early to their physical capacity and to be discharged early from the hospital. Poorly controlled pain can result in poor function with physical therapy, prolonged hospitalization, and reduced patient satisfaction.^[1]^ Therefore, adequate analgesia following TKA continues to be a topic of interest.

For a long time, femoral nerve block was the gold standard regional analgesic technique for postoperative pain treatment following TKA.^[2]^ Recently, adductor canal block (ACB) has emerged as an alternative to femoral nerve block, with the advantage of sparing the motor nerve supply to most of the quadriceps muscle and may lead to a reduction in falls after surgery.^[3,4]^ However, controversy exists regarding the ideal location of ACB placement within the adductor canal. Both distal (mid-thigh) and proximal placements have been described with each approach having benefits and drawbacks. A more proximal ACB (PACB) may offer improved analgesia and a more remote insertion site away from the surgical field; however, the proximal block may produce greater quadriceps weakness by targeting several afferent branches of the femoral nerve.^[5,6]^ The distal ACB (DACB) may have a lower risk of potentially causing quadriceps weakness, but may not provide the same level of analgesia.^[7,8]^

Several studies on this subject have been published without conclusive results.^[9–11]^ In the first randomized comparison of PACB and DACB, Mariano et al^[9]^ found that the PACB may offer better functionality during surgery without any increased quadriceps weakness. In the study by Meier et al,^[11]^ there was no difference in opioid consumption or pain scores in the first 24 hours between “proximal” versus “distal” locations for catheter placement. We thus designed a randomized controlled study to compare PACB with DACB in the treatment of TKA. It is hypothesized that no difference would be found in pain scores or opioid requirements between the 2 treatment groups. Additionally, it is hypothesized that patients receiving PACB would exhibit similar quadriceps function compared with patients receiving DACB.

## 2 Material and method

### 2.1 Study design

**Figure 1:**
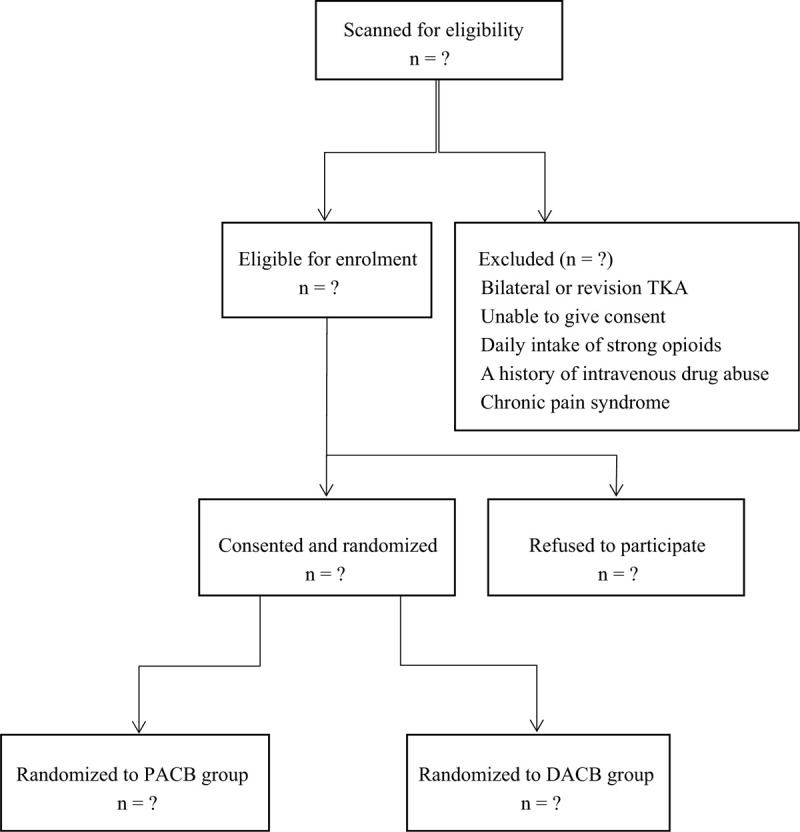
Flow diagram of the study.

This study is a single-center, prospective, assessor-blinded, randomized trial that is conducted at a single university hospital in China. Written informed consent is obtained from every participant. This report follows the Standard Protocol Items: Recommendations for Interventional Trials (SPIRIT) reporting guideline. This clinical trial was approved by the institutional review board (CRD202000104) and registered in Research Registry (researchregistry5440). The flowchart of this trial is shown in Fig. 1.

### 2.2 Eligibility criteria

From May 2020 to March 2021, adult patients ≥18 years old scheduled for primary TKA were considered eligible for the study. Patients with revision surgery, bilateral surgery, unicompartment surgery, previous enrollment in the study are excluded. Patients unable to give consent or with any known contraindications to medications, regional, or neuroaxial anesthesia are excluded. Patients with daily intake of strong opioids (morphine, methadone, fentanyl, hydromorphone), a history of intravenous drug abuse and alcohol abuse are also excluded from the study. Participants are informed that the study is comparing the efficacy of PACB and DACB for pain control following primary unilateral TKA and that they are randomly assigned to either the PACB and DACB group.

### 2.3 Randomization and blinding

We randomized consent study participants on a 1:1 ratio to 1 of 2 study groups using a computer-generated list of random numbers in varying block sizes (4–6). An investigator with no further involvement in the study generate the allocation sequence using the Web site Randomization.com, and conceal the allocation results in sealed opaque sequentially numbered envelopes that are provided to the research coordinator. On the day of surgery, and after obtaining informed consent, the research coordinator provides one envelope per patient to the anesthesiologist in the block room who perform the block procedures. The anesthesiologist who administers the block has no further role in the study; the surgeons, anesthesiologists, and nurses providing intra- and postoperative care, as well as the research coordinator assessing outcomes, are all kept blinded to allocation results.

### 2.4 Treatment protocols

For group A (PACB), the ultrasound probe is first placed for a transverse cross-sectional view of the patient's groin and thigh. The femoral nerve is identified in the short axis near the inguinal crease, and the ultrasound transducer is positioned caudally beyond the femoral triangle. We designate the position of the proximal block at the site at which the superficial femoral artery pass beneath the medial border of the sartorius muscle (generally 8–12 cm distal to the inguinal crease). These measurements, as well as the length of the thigh from inguinal crease to the top of the patella and the width of the thigh at mid-thigh, are recorded. An 18-gauge Tuohy-tip needle (Braun Medical, Melsungen, Germany) is inserted through the skin wheal and direct in-plane under ultrasound guidance. The needle is advanced toward the target until the tip of the needle crossed the sartorius muscle and pass into the adductor canal lateral to the superficial femoral artery. A 21-gauge catheter is then inserted 3 to 5 cm beyond the needle cannula. After placement, a bolus of 20 mL of 5 mg/mL ropivacaine is injected incrementally (aspiration every 3–5 mL) to expand the adductor canal space. To verify the correct position of the catheter tip, injection of an additional 1 mL of air under direct ultrasound visualization is performed. To confirm successful block, sensory function is assessed along the saphenous nerve distribution by comparing pinprick sensation to the unaffected limb. All adductor canal catheters are attached to an infusion device which infuse 2.0 mg/mL ropivacaine at a rate of 6 to 8 mL/h for at least 24 hours in all patients. The insertion site is covered with opaque silk tape to the level of the mid-thigh to ensure that postoperative assessors are blinded to the placement of the catheter.

For group B (DACB), the operative leg is exposed and measured as described above. The midpoint of the thigh is then determined as half the distance between the inguinal crease and top of the patella. After marking the mid-thigh mark with a sterile marking pen, the ultrasound transducer is positioned for a transverse view of adductor canal at mid-thigh. The femoral artery and saphenous nerve are identified under ultrasound visualization. The needle is guided through the sartorius muscle and placed lateral to the femoral artery and the saphenous nerve. Initial bolus of ropivacaine and catheter placement is performed as described above.

### 2.5 Outcomes and measures

The primary outcome of this noninferiority study is opioid consumption within the first 24 hours following surgery. Subjects receive standardized postoperative multimodal analgesics. Supplemental oxycodone 5 to 15 mg every 3 hours for pain is available to each subject. Nurses administer a 5-mg oxycodone tablet for numerical rating scale (NRS) scores of 1 to 3, 10 mg for NRS scores of 4 to 6, and 15 mg for NRS scores of 7 to 10. Oral hydromorphone is substituted for oxycodone in the instances of patient allergy or intolerance. Rescue analgesia is available with IV hydromorphone 0.5 mg every 1 hour as needed for pain of greater than NRS of 7 when refractory to oral opioids. Opioid consumption during the first 48 hours postoperatively is retrieved from the electronic medical record and convert to IV morphine equivalents for analysis. The average and worst NRS pain scores at rest and with activity are determined by blinded investigators at 24 and 48 hours postoperatively.

Secondary outcomes include quadriceps strength, pain scores, and distance ambulated, and patient satisfaction. Each subject is evaluated for maximum voluntary isometric contraction of their quadriceps muscles via force dynamometer preoperatively and at 24-hour intervals postoperatively by a blinded investigator. Subjects are given 2 attempts to produce the highest peak force, measured in pounds, during each measurement. The maximum force achieved is recorded and used for analysis. Subjects are mobilized once on postoperative day 0 and at least twice on each subsequent day with nursing or physical therapy assistance. During each physical therapy session, subjects are asked to ambulate as far as possible. The total distance, measured in feet, is recorded. Subjects are asked to give a verbal assessment representative of the quality of analgesia at 24 and 48 hours postoperatively. Response to this assessment is recorded as “satisfied” or “unsatisfied.”

### 2.6 Sample size calculation

The sample size is calculated assuming an IV morphine equivalent utilization rate of 23.7 mg with an SD of 16.9 mg within the first 24 hours following primary TKA based on prior published studies. Our noninferiority margin, 12 mg, is 50% of our known institutional opioid usage and similar to prior studies using opioid use of 10 mg as a clinically relevant difference. A noninferiority margin any smaller would have limited clinical consequence. To have a significance level of 5% and a power of 90%, 36 subjects are required in each arm. Additional subjects are recruited to prevent loss of power due to early withdrawal or protocol violations.

### 2.7 Statistical analysis

All statistical analyses are performed using SPSS v. 24 (IBM Corp., Armonk, NY). Descriptive statistics of demographic and clinical characteristics are presented with mean standard deviation for continuous scale variables. The difference between normally distributed continuous scale variables is examined using Student's *t* test, while nonnormal variables are examined using Wilcoxon rank sum test. The association between categorical variables is examined using Pearson Chi-squared test or Fisher exact test. All analyses are performed in accordance with intention-to-treat principle.

## 3 Discussion

The important opioid- and motor-sparing effects of the adductor canal block have made it an important component of multimodal analgesia for patients undergoing TKA. The adductor canal block involves the injection of local anesthetic into the adductor canal, which is a fascial compartment bordered by the sartorius, adductor, and vastus medialis muscles and traditionally described to house the saphenous nerve.^[12–14]^ Randomized clinical trials in the setting of TKA have reported that a proximal adductor canal injection location may confer superior analgesia,^[9,12]^ whereas recent cadaveric and volunteer studies propose that a distal injection location for adductor canal block should be superior because the local anesthetic not only reaches the saphenous nerve but also spreads to the popliteal plexus, thus extending analgesia to the posterior knee compartment.^[15,16]^ This randomized controlled trial aims to identify the ideal injection location for adductor canal block that optimizes postoperative analgesia and motor function after TKA.

This trial has some limitations. First, the subjects may be exclusively Chinese. Therefore, the data from this clinical trial cannot be applied to other ethnic groups. Second, owing to the small sample size, the results of this study cannot be generalized. Despite these limitations, this trial is expected to provide evidence of whether the PACB and DACB provide similar analgesia after TKA

## Author contributions

Quan Wang and Xiangjin Lin planned the study design and wrote the study protocol. Yijun Zhang and Jingyu Du reviewed the study protocol. Quan Wang, Yijun Zhang, and Xiangjin Lin will recruit participants and collect data. Quan Wang and Xiangjin Lin wrote the manuscript. All of the authors have read, commented on, and contributed to the submitted manuscript.
